# A performance evaluation of commercially available and 3D-printable prosthetic hands: a comparison using the anthropomorphic hand assessment protocol

**DOI:** 10.1186/s42490-024-00086-w

**Published:** 2024-12-02

**Authors:** Joshua R. Siegel, Jedidiah K. Harwood, Annette C. Lau, Dylan J. A. Brenneis, Michael R. Dawson, Patrick M. Pilarski, Jonathon S. Schofield

**Affiliations:** 1 Department of Mechanical and Aerospace Engineering, University of California, Davis, Davis, CA USA; 2 Department of Statistics, University of California, Davis, Davis, CA USA; 3 Department of Medicine, University of Alberta, Edmonton, AB Canada; 4 Alberta Machine Intelligence Institute (Amii), Edmonton, AB Canada; 5 Department of Mechanical Engineering, University of Alberta, Edmonton, AB Canada

**Keywords:** Hand prosthesis, 3D-printed prosthetics, Prosthetics, Upper-limb prosthetics, Upper-limb prosthetic evaluation, Commercially available hand prostheses

## Abstract

Despite significant technological progress in prosthetic hands, a device with functionality akin to a biological extremity is far from realization. To better support the development of next-generation technologies, we investigated the grasping capabilities of clinically prescribable and commercially available (CPCA) prosthetic hands against those that are 3D-printed, which offer cost-effective and customizable solutions. Our investigation utilized the Anthropomorphic Hand Assessment Protocol (AHAP) as a benchtop evaluation of the multi-grasp performance of 3D-printed devices against CPCA prosthetic hands. Our comparison sample included three open-source 3D-printed prosthetic hands (HACKberry Hand, HANDi Hand, and BEAR PAW) and three CPCA prosthetic hands (Össur i-Limb Quantum, RSL Steeper BeBionic Hand V3, and Psyonic Ability Hand), along with including previously published AHAP data for four additional 3D-printed hands (Dextrus v2.0, IMMA, InMoov, and Limbitless). Our findings revealed a notable grasping performance disparity, with 3D-printed prostheses generally underperforming compared to their CPCA counterparts, specifically in cylindrical, diagonal volar, extension, and spherical grips. We propose that the observed performance shortfalls are likely attributed to the design or build quality of the 3D-printed prostheses, owing to the fact that 3D-printed hands often have a lower technology readiness level for widespread use. Addressing the limitations highlighted in this work and subsequent research will play a crucial role in refining the design and functionality of both 3D-printed and CPCA prosthetic devices.

**Supplementary Information:**

The online version contains supplementary material available at 10.1186/s42490-024-00086-w.

## Background

The human hand is a sophisticated biological machine that allows individuals to perform a range of activities, from delicate precision grasping tasks to robust and powerful gripping actions. Consequently, upper-limb loss not only interferes with a person’s ability to perform activities of daily living, but may also trigger a broad range of emotional and psychological problems, including anxiety, depression, and post-traumatic stress disorder [1–3]. Although there may be some uncertainty in long term predictions, the increasing prevalence of upper-limb difference, with estimates suggesting 540,000 patients currently in the United States and an annual addition of 30,000 cases, demands urgent and advanced progress in prosthetic technologies [4, 5].

Despite centuries of work towards the creation of artificial limbs, the development of a device whose function closely approaches that of a biological extremity is far from realized. Abandonment rates of upper-limb prostheses remain high, with an estimated 44% of individuals living with limb difference choosing to discontinue using a prosthesis [6]. Additive manufacturing, or 3D printing, began to influence the field of prosthetics in 2012, when the first 3D-printed prosthesis, “Robohand”, was introduced [7]. Since 2012, the field has continuously evolved, bringing to market a variety of open-source 3D-printed prosthetic models. This method significantly reduces production costs, with 3D-printed prostheses starting as low as $19USD in raw materials and parts, in stark contrast to clinically prescribable and commercially available (CPCA) devices that can often cost upwards of $20,000USD [8–10]. The affordability and accessibility of 3D printing have also made prosthetics research more inclusive, enabling studies across labs with varying funding levels and promoting a diverse research ecosystem. This technology not only facilitates rapid prototyping, allowing for the swift design, production, and testing of prosthetic components but also supports the customization of designs to meet individual anatomical needs and preferences. Such flexibility is key from a research and design perspective when exploring prosthetic functionality and enhancing user comfort. Despite these advancements, 3D printing technology in prosthetics is still maturing, and comprehensive research into the dexterity and functionality of 3D-printed prosthetic hands, as juxtaposed with CPCA prostheses, remains in the nascent stages.

Previous research in prosthetics has often been compartmentalized, with studies typically focusing on either 3D-printed or CPCA prosthetic hands in isolation. For example, Llop-Harillo et al. and Cabibihan et al. performed work to evaluate the performance of a variety of 3D-printed hands [9, 11]. In contrast, Belter et al. and Kannenberg et al. have independently explored the efficacy of CPCA prosthetic hands [12, 13]. The varied directions taken in prosthetics research have created a significant knowledge gap concerning the systematic evaluation and benchmarking of both 3D-printed and CPCA prostheses within the same standardized testing framework. Our study aims to fill this void and contribute to this evolving landscape by conducting an evaluation that benchmarks the mechanical grasping abilities of both CPCA and 3D-printed prosthetic hands. This evaluation focuses on tasks involving physical objects that users are likely to encounter in their daily activities, explicitly assessing machine-to-object interaction.

## Methods

### Testing methods

**Fig. 1:**
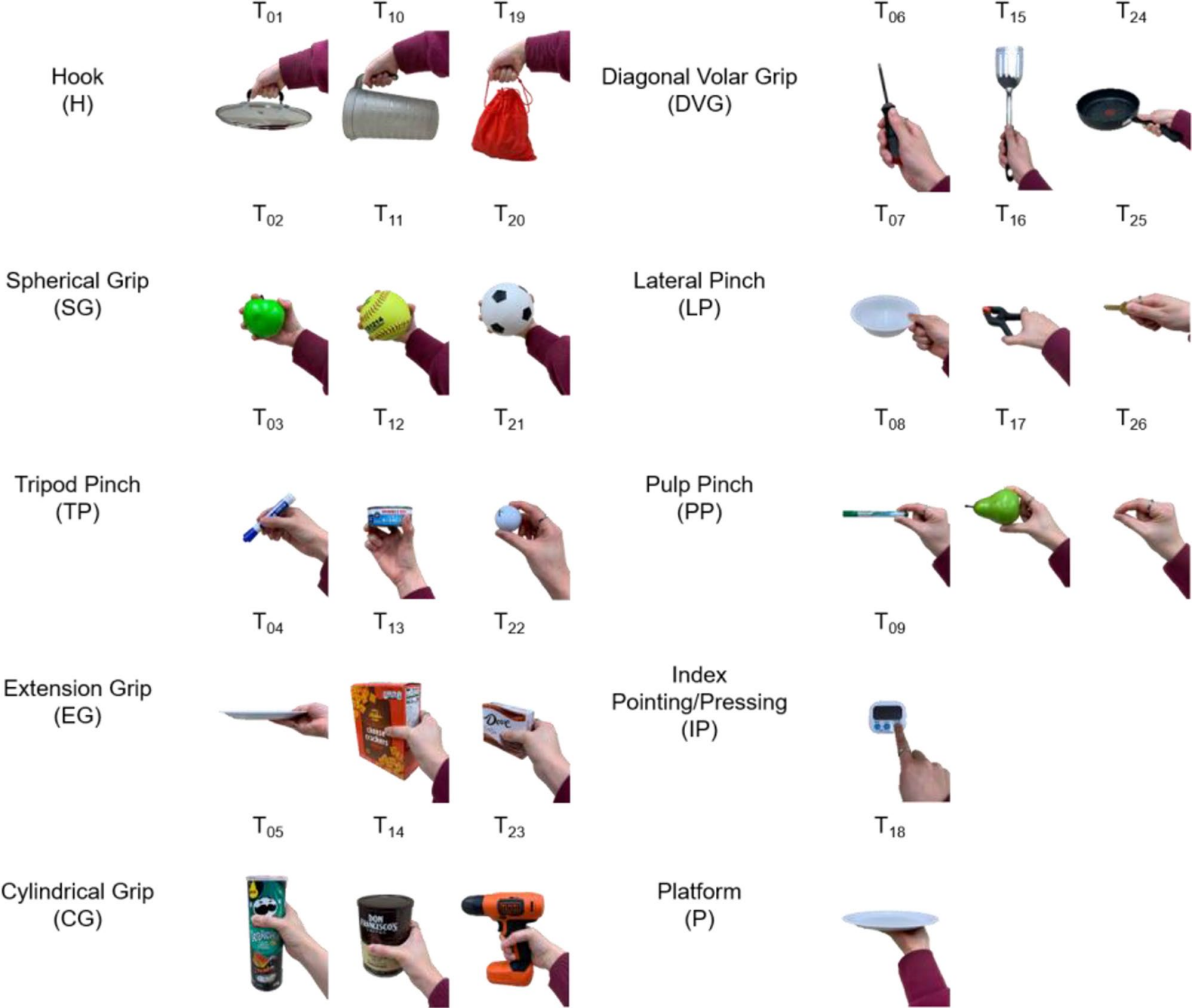
AHAP tasks and grips

The Anthropomorphic Hand Assessment Protocol (AHAP) was the evaluation metric we employed [14]. This validated procedure was developed by others as a benchtop test in which common household items are grasped and held. It is used to assess the mechanical grasping capabilities of dexterous prosthetic hand offering multiple griping configurations and is designed to be performed independent of a prosthesis user. That is, the hand itself is being evaluated without possible confounding variables such as the choice of control system, prosthetic socket fit, or the user’s skill level in controlling their device, among many others. The AHAP involves 26 specific tasks using 25 household objects and encompasses 10 different grip patterns: hook grip, spherical grip, tripod pinch, extension grip, cylindrical grip, diagonal volar grip, lateral pinch, pulp pinch, index pointing/pressing, and platform [14]. The AHAP tasks and grips are shown in Fig. 1. The AHAP’s reliability is notable, scoring a test-retest reliability intraclass correlation coefficient (ICC) of 0.839, an inter-rater reliability ICC of 0.969, and an internal consistency Cronbach’s alpha of 0.846 [14, 15]. For our analysis, we included three open-source 3D-printed prosthetic hands—HACKberry Hand, HANDi Hand, and BEAR PAW—and three frequently prescribed, CPCA prosthetic hands—Össur i-Limb Quantum, RSL Steeper BeBionic Hand V3, and Psyonic Ability Hand. Additionally, the AHAP results from these six hands were then combined with previously published AHAP scores from four additional 3D-printed hands: Dextrus v2.0, IMMA, InMoov, and Limbitless [14].

Execution of the AHAP followed the established protocol described in [13]. This involved a lead investigator who conducts the testing and scoring, and three test investigators who are responsible for operating the prosthesis. Each test investigator performed three trials, for a total dataset of nine trials per prosthesis (3 test investigators x 3 trials each = 9 total trials). The standard AHAP procedure required replicating the test with multiple trials and three separate test investigators to ensure the results were isolated to only characterize the mechanical performance of the tested prosthesis by accounting for potential variability across investigators [14]. Before starting the testing protocol, the lead investigator briefed the test investigators on the proper grip type for each object and allowed a one-minute familiarization period. All tested prostheses were mounted on a wand and operated by selecting a grasp from a list (using a button or graphical interface on a tablet, dependent on the design of the prosthesis) and actuating the grasp by pressing a button, thus eliminating the potential impacts in the choice of and skill in using a conventional prosthetic control system (such as 2-site electromyography, pattern recognition, etc.). Each AHAP trial commenced with the lead investigator presenting one of the 26 objects to a test investigator in a specific orientation. For each grip type (except index pointing/pressing), the prosthesis was initially positioned with the palm facing upwards. Upon securing the object, the prosthesis was required to sustain its grip on the object for a duration of three seconds (the grasping phase). This was followed by a 180° pronation to a palm-down position (clockwise for left-handed prostheses and anti-clockwise for right-handed prostheses), again trying to maintain its grip for an additional three seconds (the maintaining phase). Further descriptions of the grasping and maintaining phases for each grip type and posture can be found in the original AHAP instructions by Llop-Harillo et al. [14].

Following the AHAP protocol [14], during the grasping and maintaining phases for each object the lead investigator scored the prosthesis’s performance. Accordingly, a score of 1 was received if the object was held with the specified grip for the allotted time. A score of 0.5 was given if the prosthesis held the object for the designated time but did not follow the specific grip requirements described by the AHAP. Finally, a score of 0 was received if the prosthesis was unable to hold the object at all. Then, if there was no movement of the object within the hand during the maintaining phase, a score of 1 was awarded. If the object moved but did not drop, then a score of 0.5 was received, and a score of 0 was given if it was not able to maintain the object. A score of 0 may also be assigned at the lead investigator’s discretion, without attempting the grip, when it was deemed likely that an attempt would cause functional damage to the hand.

Scores were separated by phase: grasping or maintaining. The scores for each prosthetic hand were further separated into 10 categories for grasping and nine categories for maintaining classified by grip type/posture. These scores were averaged across the three test investigators such that individual grasping and maintaining comparisons could be made between hands. Finally, an overall grasping ability score (GAS) was given for each hand by averaging all scores. Each prosthesis’s raw AHAP scores are provided in the supplementary material.

#### Hands tested

We have provided a brief description for each of the six tested hands below. Technical data has also been summarized in Table 1. Data in this table were tested and published by the respective manufacturers of each hand.

**Table 1:**
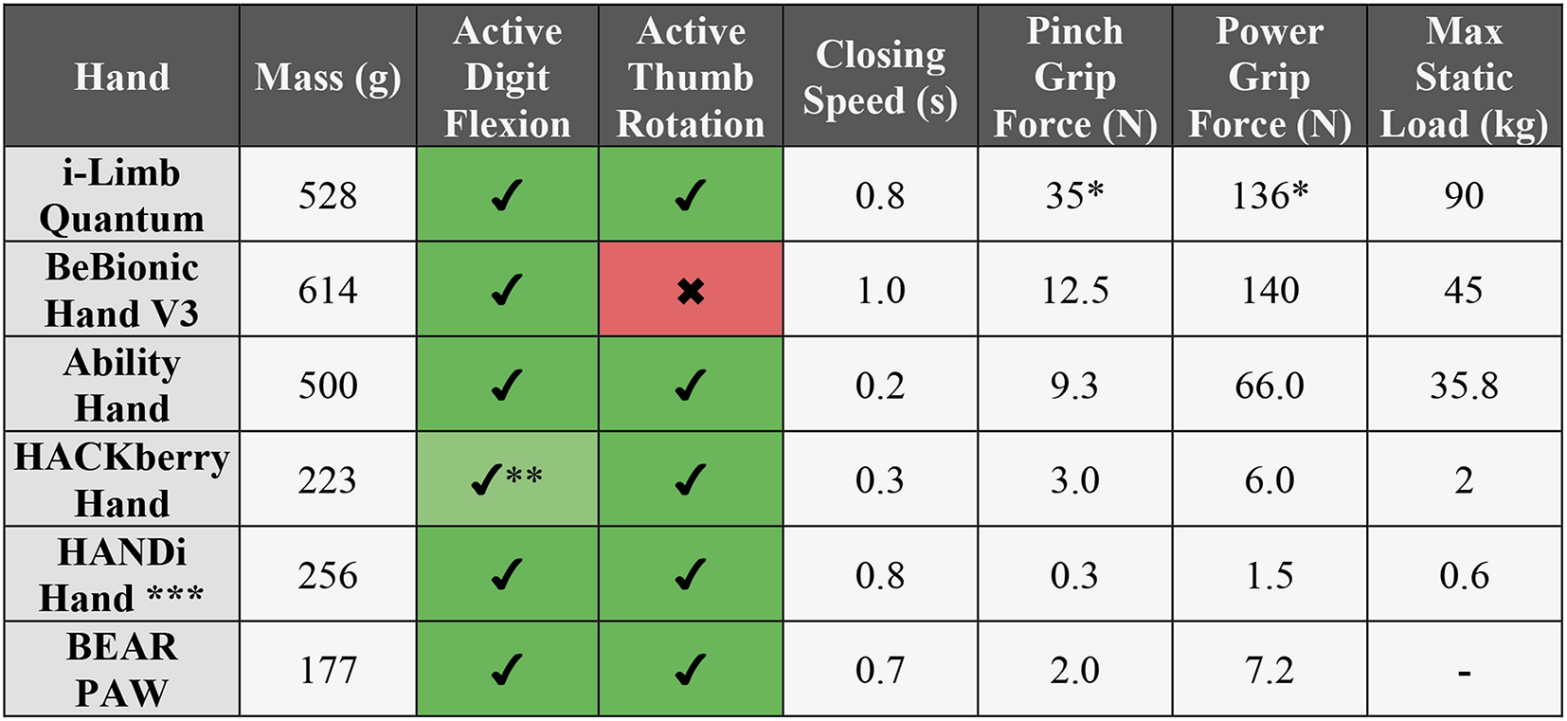
Technical data of the six tested prosthetic hands

*Grip forces are no longer stated in spec sheets for the i-Limb Quantum so these values are estimated from the similar i-Limb Ultra Revolution model [15]

** For HACKberry hand: 1st motor flexes index, 2nd motor flexes D3-D5, and Thumb flexion is passive

*** For HANDi Hand: used Dymond D47 servo motors instead of the original Hitec HS35-HD motors

**Fig. 2:**
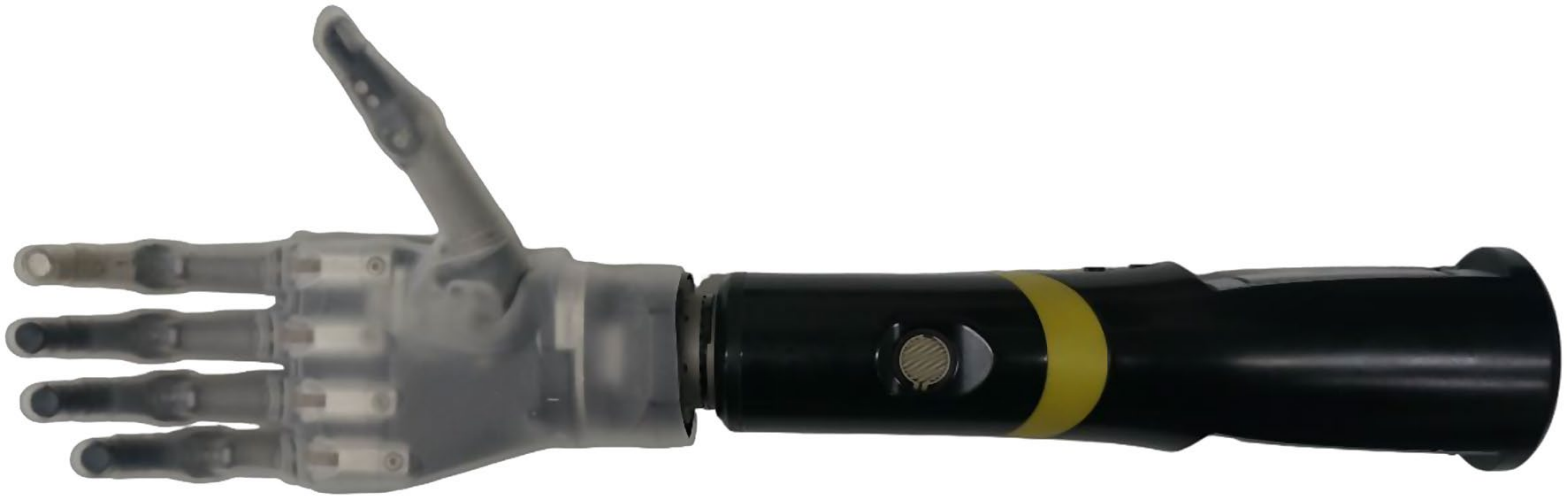
Össur i-Limb quantum

***Össur i-Limb Quantum***

The Össur i-Limb Quantum (shown in Fig. 2) [16–18] is a CPCA prosthetic hand. Built with titanium digits, the i-Limb Quantum weighs 658 g, has a static limit finger carry load of 48 kg and a hand load static limit of 90 kg. It is equipped with five independently motorized fingers and a powered thumb rotation with manual override. Using the My i-Limb™ iOS apps, it has up to 36 selectable grips, both pre-programmed and customizable.

***RSL Steeper BeBionic V3***

**Fig. 3:**
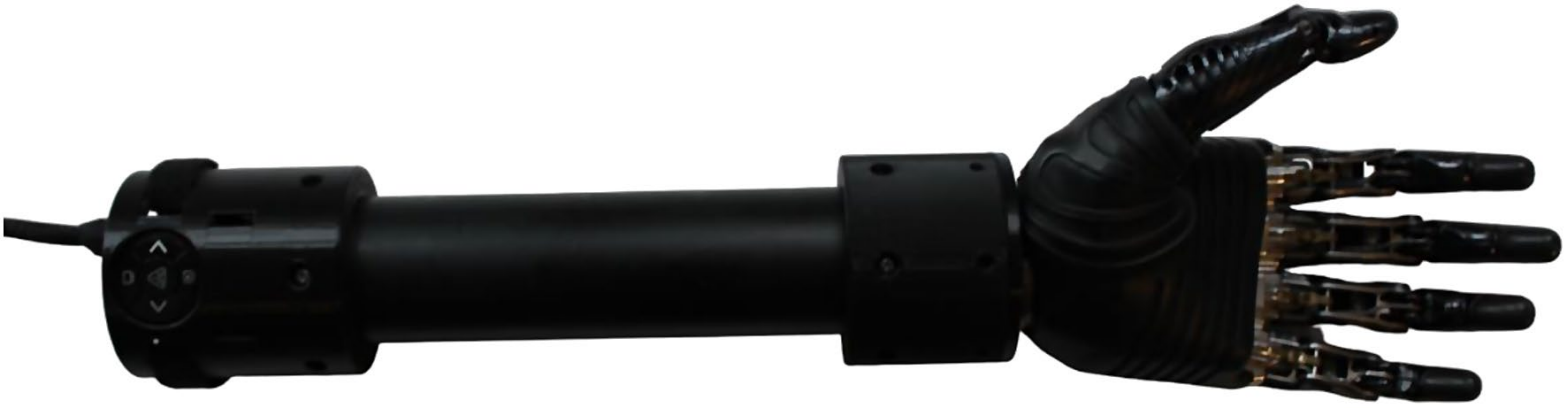
RSL steeper BeBionic V3

The RSL Steeper BeBionic Hand V3 (shown in Fig. 3) [19–21] has been commonly prescribed as a hand prosthesis since 2010 [19]. Built with carbon fiber digits, the BeBionic V3 weighs 588 g, has a static limit finger carry load of 25 kg and a hand load static limit of 45 kg. The hand’s individual motors located in each finger and at the thumb base enable the user to have five degrees of actuation (passive thumb rotation) and 14 different grips and hand positions [19].

***Psyonic Ability Hand***

**Fig. 4:**
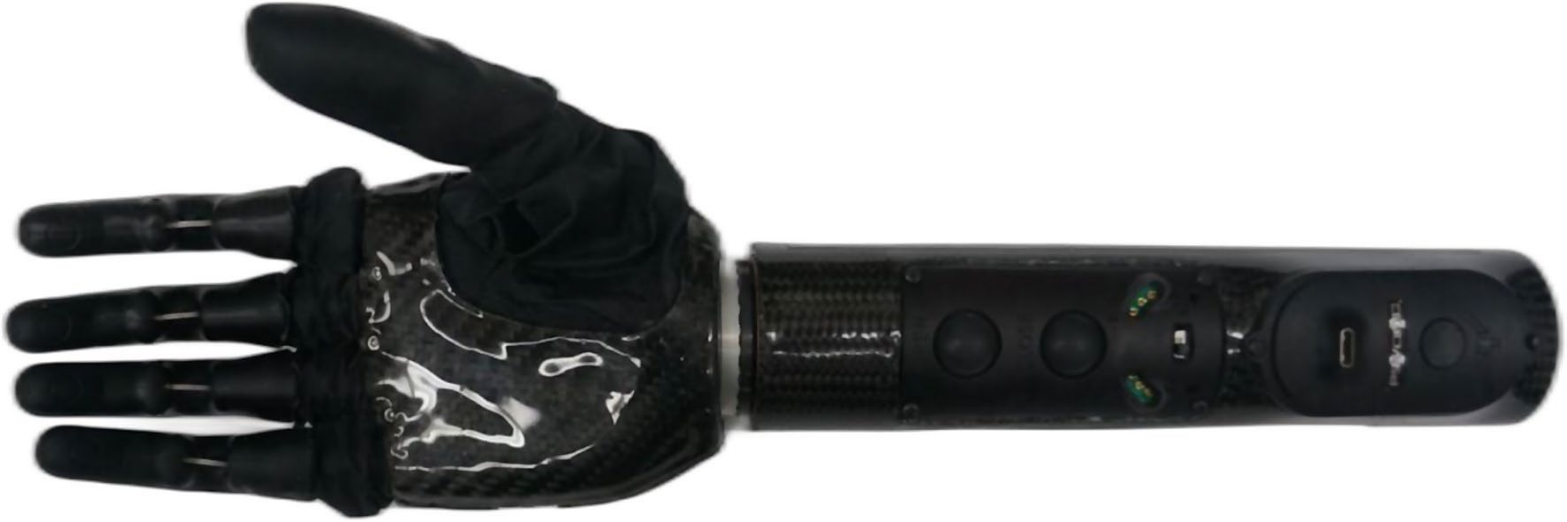
Psyonic ability hand

The Psyonic Ability Hand (shown in Fig. 4) [22, 23] is a CPCA prosthetic hand. It is equipped with fingertip sensors that detect pressure during gripping and send vibrations to the user’s arm, offering some tactile feedback. With its carbon fiber shell, the Ability Hand weighs 520 g and has a maximum grip force of 66 N. The Ability Hand has five independently motorized fingers and a powered thumb rotation with manual override. The hand comes pre-programmed with 32 grip patterns, including 19 predefined options. The Ability Hand is compatible with third-party EMG pattern recognition systems, EMG direct control systems, linear transducers, and force-sensitive resistors along with integration with iOS and Android mobile apps for adjustment of settings and updates.

***HACKberry Hand***

**Fig. 5:**
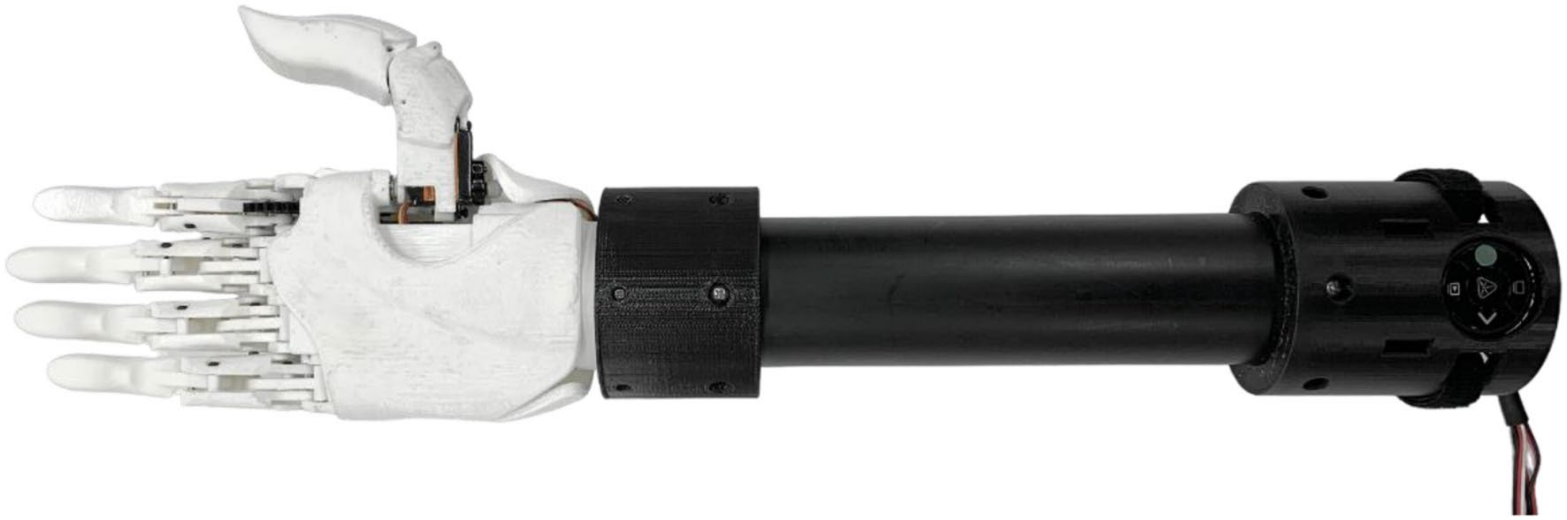
HACKberry hand

The HACKberry Hand (shown in Fig. 5) [24], developed by Japanese startup Exiii, is an open-source 3D-printable bionic prosthetic hand. The HACKberry hand is made of polylactic acid (PLA), weighs 475 g, and can support loads up to 2000 g. Equipped with three motors, it has partially motorized long fingers and powered thumb rotation. The third, fourth, and fifth fingers are coupled, allowing them to flex and extend as a group. Additionally, it has passive thumb flexion, allowing it to meet the index finger for a pinch grip. When attached to its arm component, it has passive wrist flexion and rotation. The HACKberry hand does not come with pre-programmed grips, however they can be defined at the user’s discretion so long as it remains within the range of motion of the finger joints.

***HANDi Hand***

**Fig. 6:**
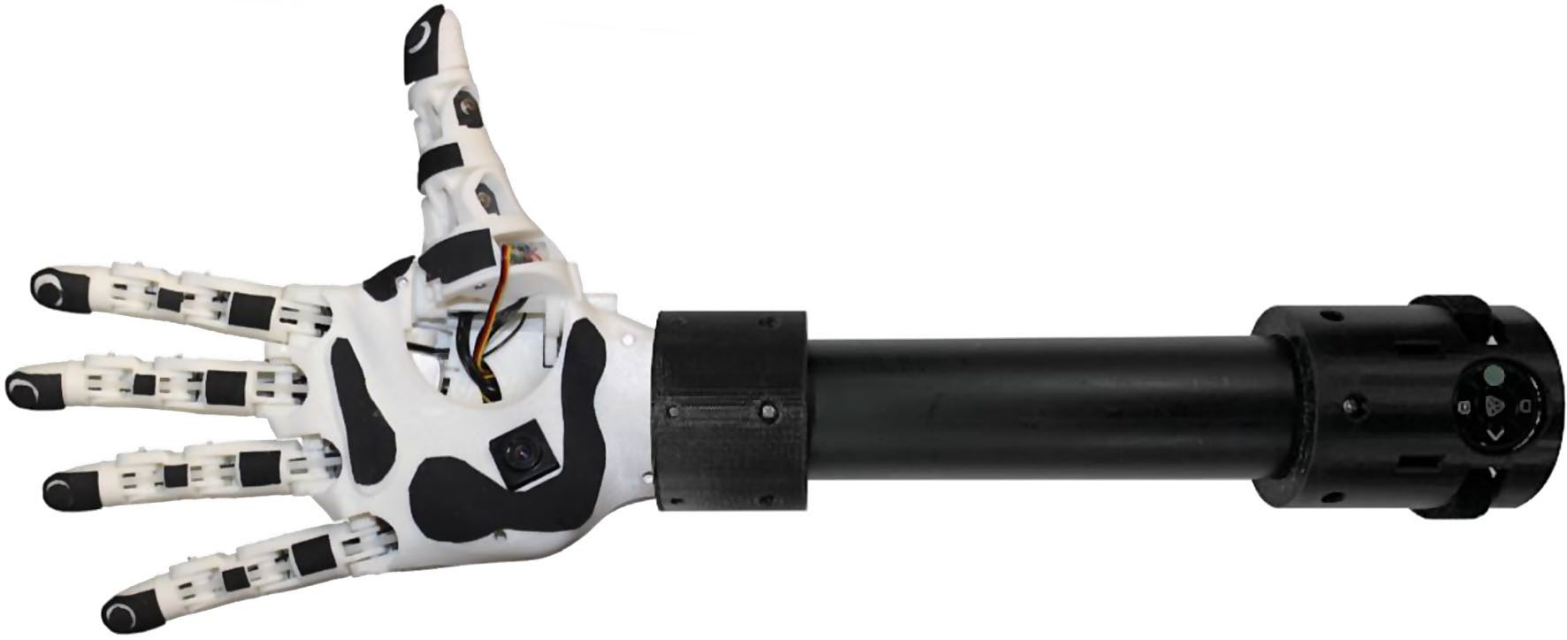
HANDi hand

The Humanoid, Anthropometric, Naturally Dextrous Intelligent (HANDi) Hand (shown in Fig. 6) [25] is a 3D-printed multi-articulating hand. It was developed at the Bionic Limbs for Improved Natural Control Laboratory (BLINC Lab). The hand can be used in conjunction with the Bento Arm, a five-degree-of-freedom robotic arm designed for myoelectric training and research applications [26]. Made of PLA, the HANDi Hand weighs 256 g and has a maximum grip force of 4.2 N. Six integrated Dymond D47 servo motors allow for individual finger articulation, with separate thumb rotation and flexion. Rotary potentiometers in the joints and force-sensitive resistors in the fingertips can provide finger position and force information to machine learning algorithms. Further, a USB webcam is integrated into the palm, providing visual information about the hand’s workspace. The HANDi hand must be programmed by the user as it does not come with pre-programmed grips.

***BEAR PAW***

**Fig. 7:**
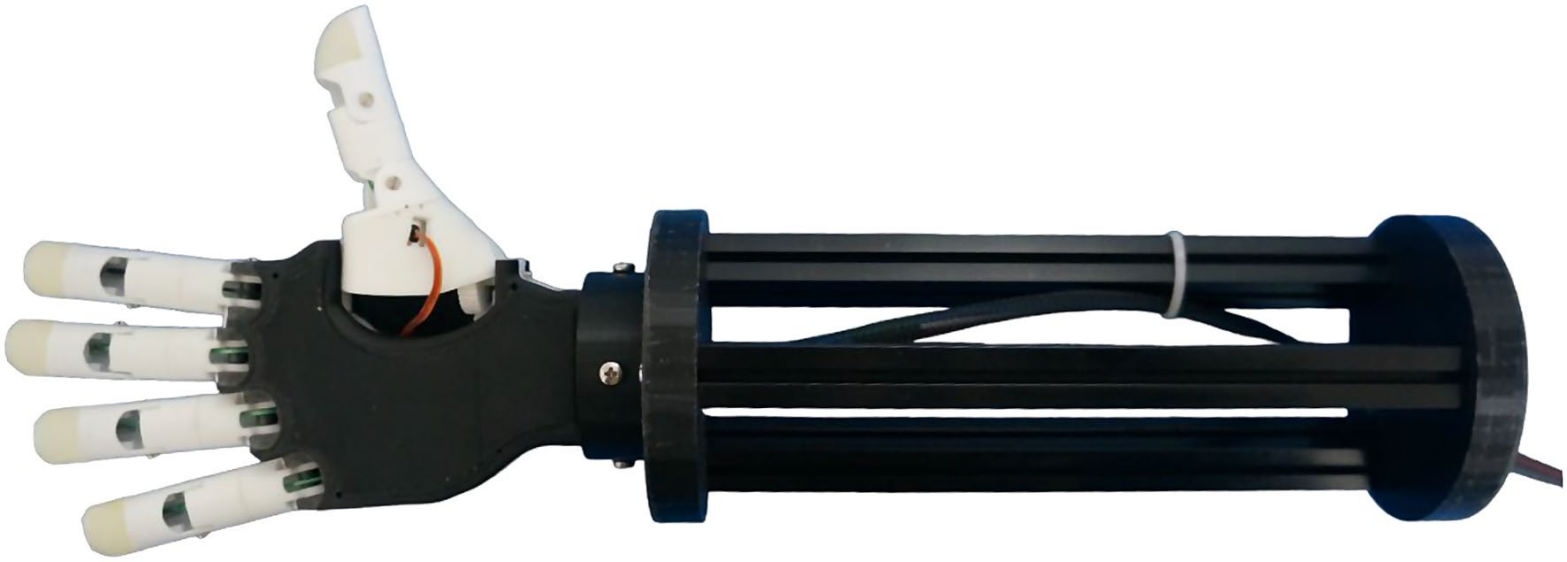
BEAR PAW

The Bionic Engineering and Assistive Robotics Pediatric Assistive Ware (shown in Fig. 7) (BEAR PAW) is a 3D-printed pediatric prosthetic hand developed at the UC Davis Bionic Engineering and Assistive Robotics Laboratory (BEAR Lab) [27]. Modeled after the anatomical proportions of an 8-year-old child and printed using PLA, it has a weight of 177 g with a maximum grip force of 7.2 N. The BEAR PAW has five independently motorized fingers and a powered thumb rotation. The hand comes pre-programmed with 10 grip patterns, and additional grips can be created by the user.

***Dextrus v2.0***,*** IMMA***,*** InMoov***,*** and Limbitless***

Llop-Harillo et al. published AHAP data for four open-source adult 3D-printed hands (Dextrus v2.0, IMMA, InMoov, and Limbitless) [11]. Printed either using PLA or Ninjaflex^®^, they ranged in weight from 131 g to 201.5 g but did not publish grip force or static load values. These four adult hands were all underactuated systems with a range from 14 to 17 degrees of freedom and 1–6 degrees of actuation. The testing for these hands was done using the custom-made Able-Bodied Adapter presented by Llop-Harillo and Pérez-González in 2017 [28].

### Data analysis

**Fig. 8:**
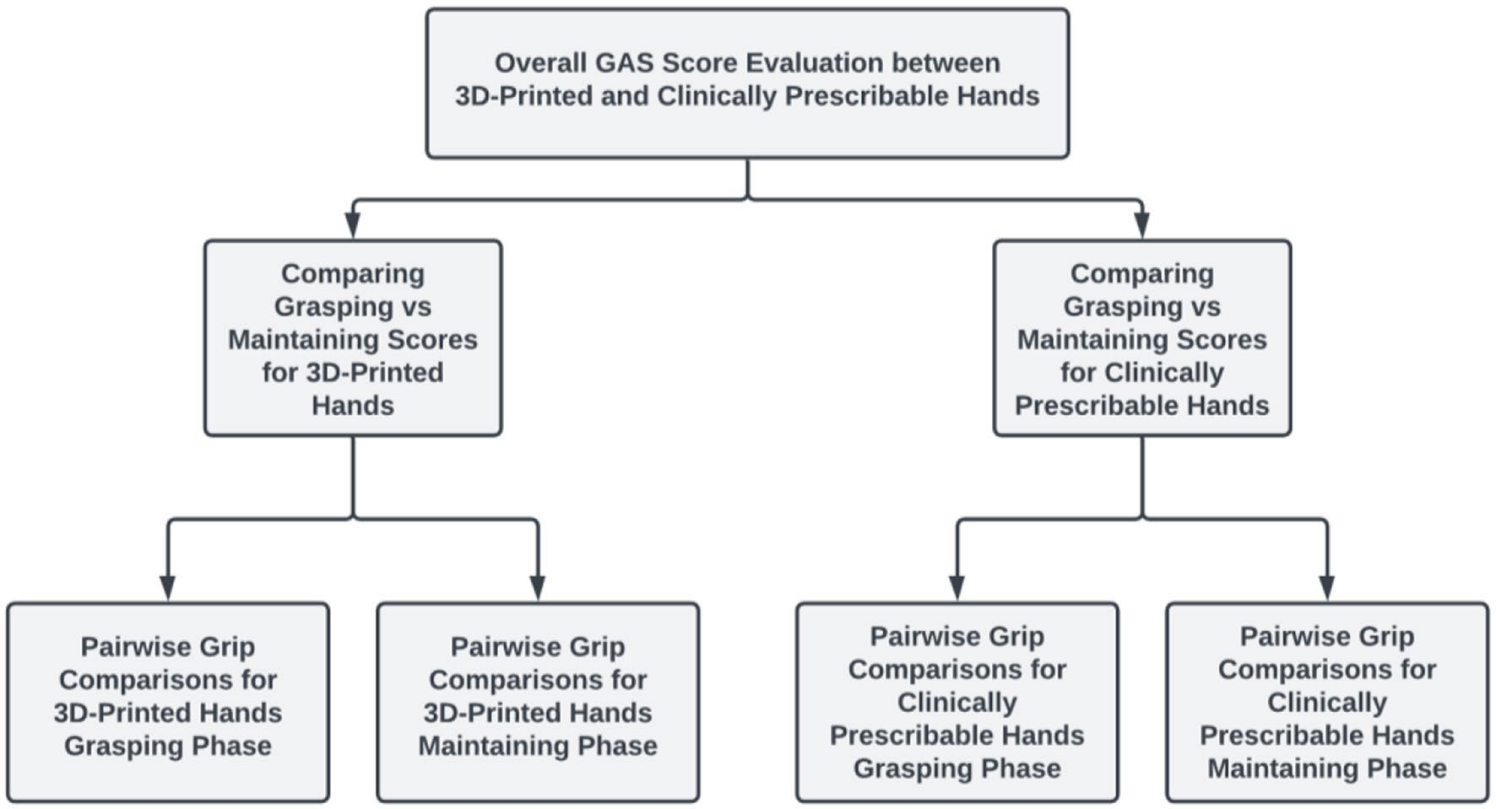
Statistics logic flow diagram

Our data analysis was designed to allow for the group-based comparison of overall grasping scores between the 3D-printed and CPCA hands, followed by an assessment of grasping versus maintaining scores within these specific groups. Subsequently, we conducted pairwise grip comparisons within each subgroup. This procedure is depicted in Fig. 8, with further descriptions of each comparison provided below.

#### Overall GAS score evaluation

In our investigation, we utilized the Mann-Whitney U-Test to compare the overall performance (GAS scores) between CPCA and 3D-printed prosthetic hands. The Mann-Whitney U-Test was chosen to accommodate our small sample size, non-parametric data, and to fit our assumption of a bimodal distribution, as we could not assume a normal distribution for the GAS scores [29]. The Mann-Whitney U-Test operates by converting actual data points into ordered ranks to form a permutation distribution, from which it calculates the p-value [29]. We also confirmed, using our data characteristics, that the prerequisites for the Mann-Whitney U-Test were met [29]. This included ensuring that the observations within and across groups were independent and that our response variable was ordinal or continuous [29].

#### Comparing grasping vs. maintaining scores

We also investigated whether there were differences between grasping and maintaining scores for both the 3D-printed and CPCA hands as individual groups. To do this, we conducted two additional Mann-Whitney U-Tests. In consideration of conducting a series of related tests, we employed the Bonferroni Correction to keep the family-wise error rate below 5%. Consequently, the inclusion of these two Mann-Whitney U-Tests, alongside the overall GAS score comparison, established a test-wise significance threshold of 1.67% [30].

#### Pairwise grip comparisons

Finally, we wanted to determine which specific grips the hands may have struggled with. To do so, we utilized Friedman’s Test (a non-parametric alternative to a Two-Way ANOVA model without an interaction term), followed by the Nemenyi Test (if applicable) [31, 32]. Similar to the Mann-Whitney U-Test, Friedman’s Test works by converting the data into ordered ranks and using a permutation distribution to calculate the p-value (indicating if there were differences among grips at a significance threshold of 5%). The Nemenyi Test, designed as a non-parametric pairwise comparison method, enabled the identification of specific grip patterns that exhibited lower performance, provided that Friedman’s Test indicated significant differences [31].

## Results

### AHAP results

Table 2 presents the average overall GAS, grasping, and maintaining scores along with their standard deviations for each prosthetic hand, as measured using the AHAP. The scores in Table 2 are expressed as a percentage of the maximum achievable score [11].

**Table 2:**
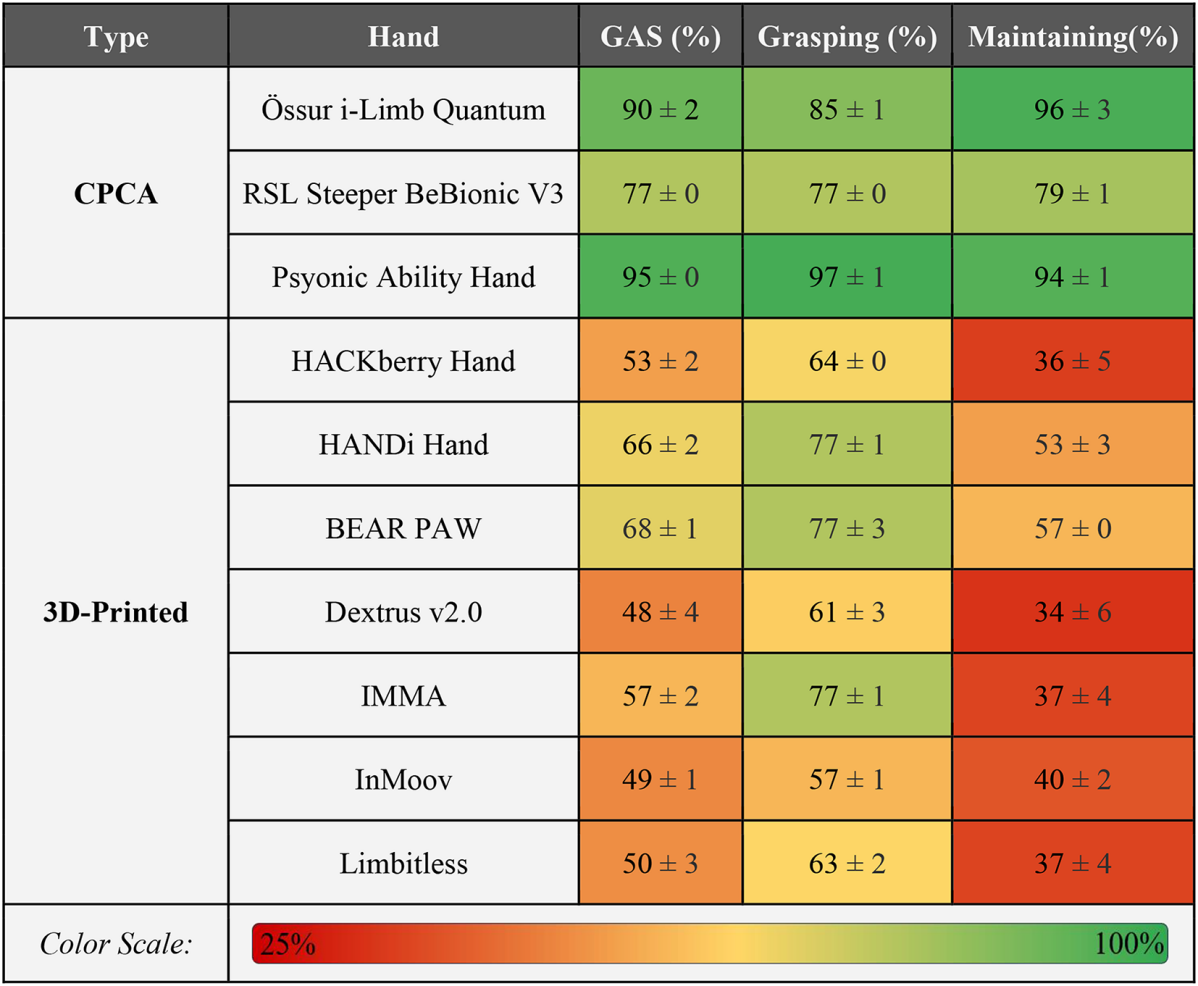
AHAP scoring results

### Data analysis results

#### Overall GAS score evaluation

**Fig. 9:**
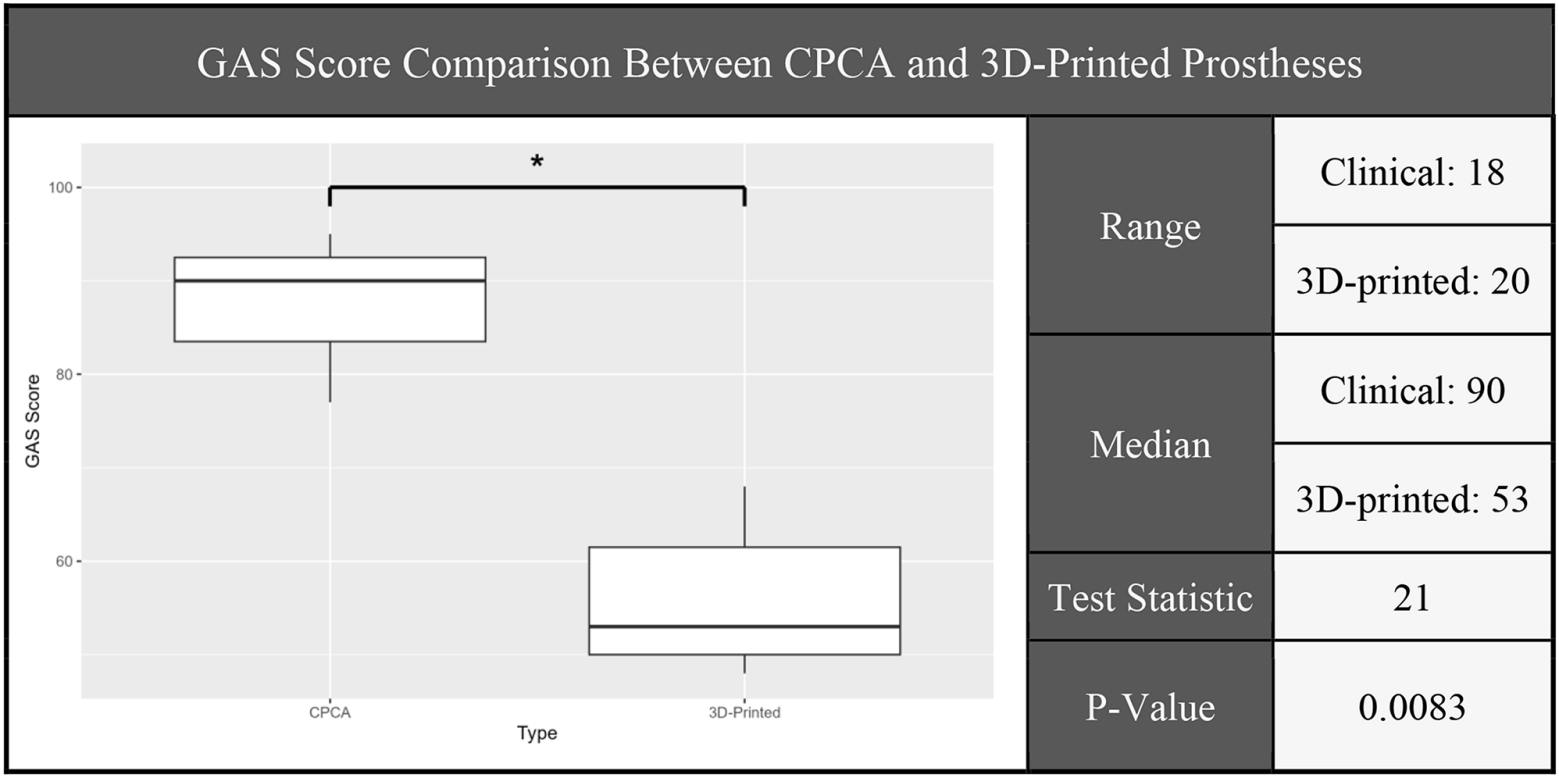
Overall GAS score Mann-Whitney U-test results: CPCA hands significantly outperformed 3D-printed

Our analysis indicates a statistically significant difference in overall GAS scores between the CPCA and 3D-printed prostheses, with data inspection indicating lower performance by the 3D-printed prostheses. Our initial assumption under the null hypothesis was that both groups of prostheses (3D-printed and CPCA) showed identical underlying distributions. Figure 9 displays the Mann-Whitney U-Test results, including a P-value of 0.0083. This permitted rejecting the null hypothesis using a test-wise significance level of 1.67% (post Bonferroni Correction).

#### Comparing grasping vs. maintaining scores

**Fig. 10:**
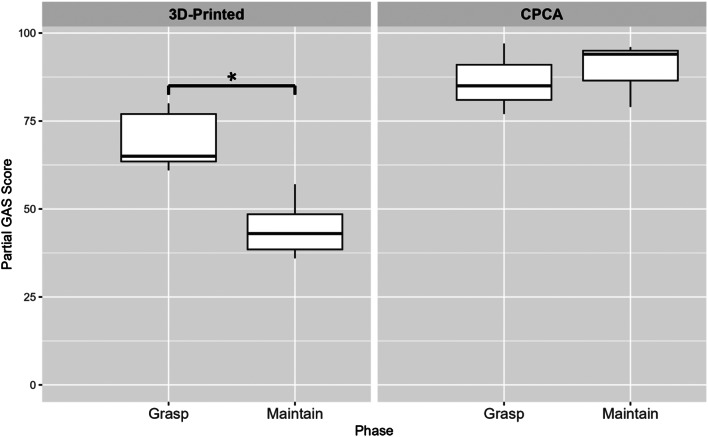
Comparing grasping vs. maintaining scores: 3D-printed hands particularly struggled during the maintaining phase of the AHAP. CPCA hands had consistent results between grasping and maintaining

The outcomes of these analyses demonstrated statistically significant disparities within the 3D-printed hands, also at a test-wise significance level of 1.67%, indicated by a P-value of 0.0011 and a test statistic of 49. Conversely, the comparisons within CPCA prosthetic hands did not reveal statistical significance, as shown by a P-value of 0.50 and a test statistic of 4. The findings of these tests are detailed in Fig. 10. For these tests, the null hypothesis assumed that the scores for grasping and maintaining were identical within each type of prosthetic hand—first examining this assumption for 3D-printed hands, and then separately for CPCA hands.

#### Pairwise grip comparisons

##### Friedman’s test

**Fig. 11:**
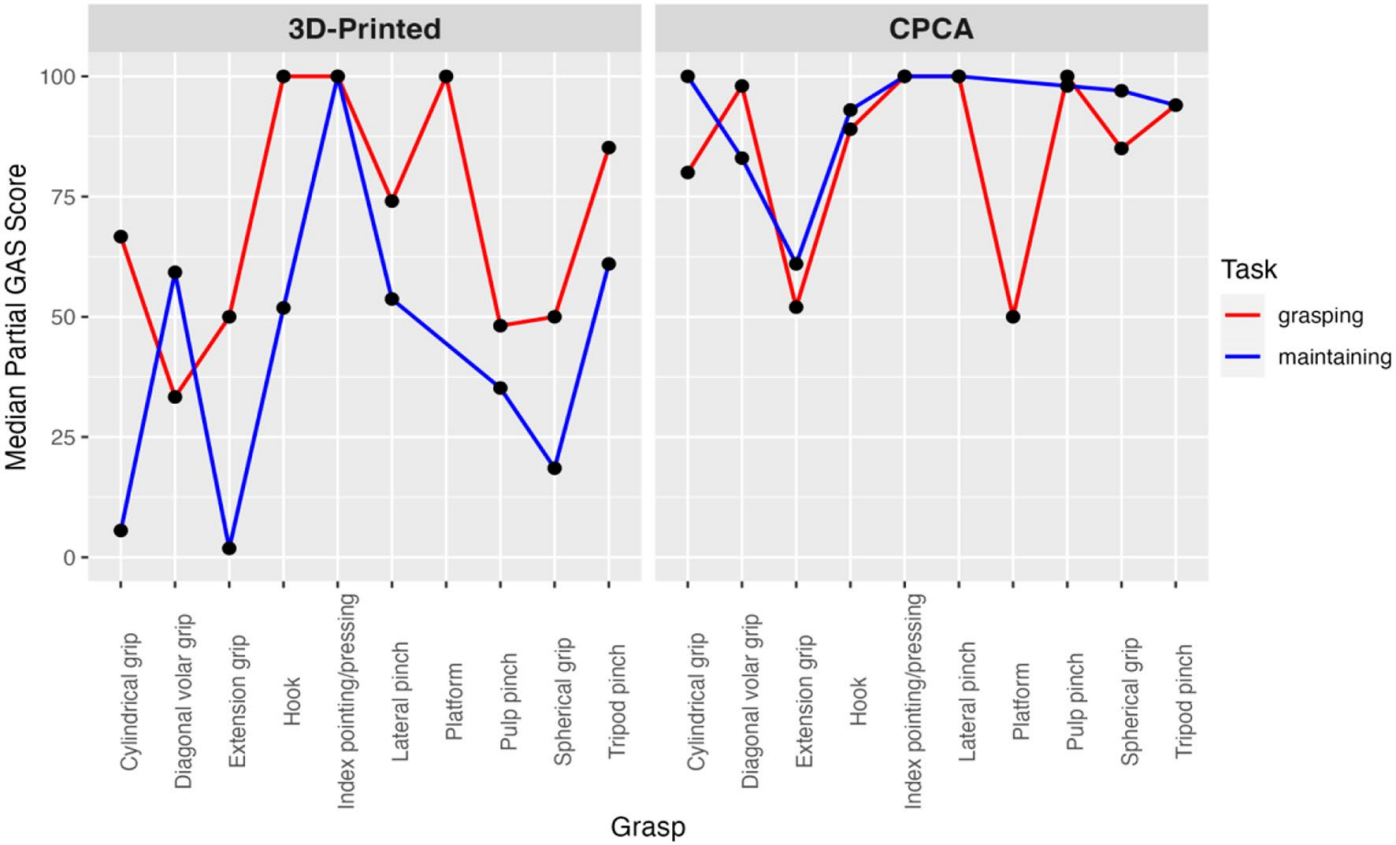
Grip-wise comparison using Friedman’s test: 3D-printed hands struggled with certain grips during both the grasping and maintaining phase. CPCA hands only struggled with certain grips during the grasping phase

Figure 11 presents the outcomes of the Friedman’s Tests, highlighting statistically significant differences in the grip patterns within both the grasping and maintaining scores for the 3D-printed prosthetic hands, with P-values of 4.11e-05 and 1.14e-05, and test statistics of 35.91 and 37.03, respectively. For the CPCA prosthetic hands, the results demonstrated statistically significant differences within the grasping scores across different grips, as indicated by a P-value of 0.033 and a test statistic of 18.16. However, the maintaining scores of CPCA hands did not exhibit significant variations across grip types, evidenced by a P-value of 0.24 and a test statistic of 10.45.

##### Nemenyi test for 3D-printed hands

**Fig. 12:**
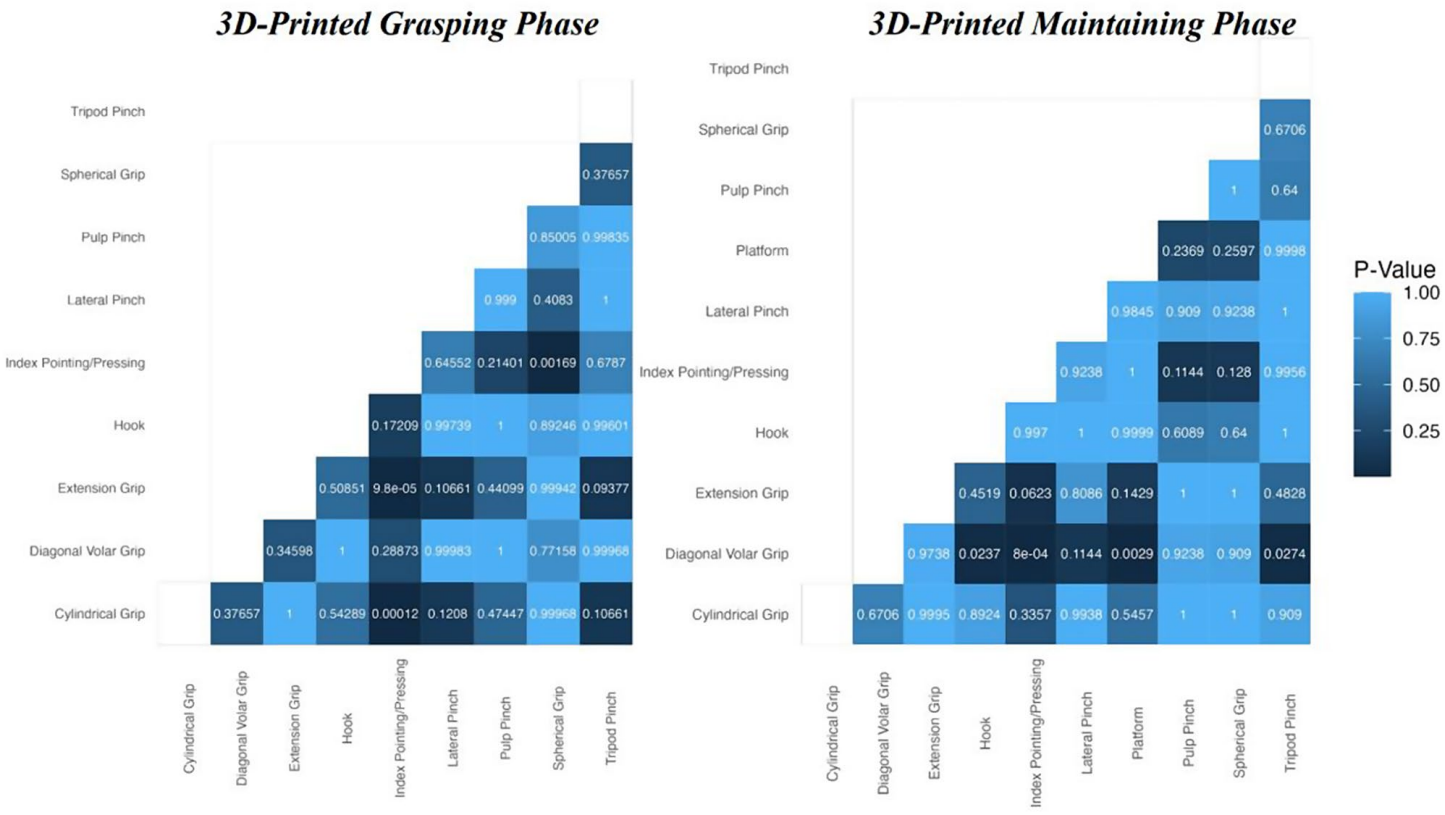
Specific pairwise comparison using the Nemenyi test showed the 3D-printed hands struggled with diagonal volar grip during the grasping phase along with the cylindrical grip, extension grip, and spherical grip during the maintaining phase

The Nemenyi Test results highlighted significant disparities in the grasping scores of the 3D-printed hands and can be found in Figs. 11 and 12. Specifically, the Diagonal Volar grip demonstrated considerable differences compared to the Hook, Index Pointing/Pressing, Platform, and Tripod Pinch grips. Additionally, for maintaining scores, the Index Pointing/Pressing grip was significantly different from the Cylindrical Grip, Extension Grip, and Spherical Grip. For the other grip types evaluated within the 3D-printed hands, there were no statistically significant differences.

##### Nemenyi test for CPCA hands

Unfortunately, while Friedman’s Test indicated statistically significant differences among the grasping scores for the CPCA hands, the subsequent Nemenyi Test P-values were statistically insignificant. Therefore, we could not specify which grip pairs exhibited these differences. However, inspection of Fig. 11 suggests that the Extension and Platform grips underperformed compared to other grips. Finally, because Friedman’s Test was unable to show differences among the maintaining scores of the CPCA hands, we excluded them from the Nemenyi Test analysis.

## Discussion

Despite the evolving landscape of CPCA and 3D-printed multi-grasp prosthetic hands little literature exists to examine the mechanical performance these two groups of hands using the same standardized testing protocol. Our AHAP analysis of the mechanical grasping abilities of CPCA and 3D-printed prosthetic hands, highlighted significant differences in their performance. Specifically, it was observed that the 3D-printed prosthetic hands had lower scores compared to their CPCA alternatives. Furthermore, the performance gap observed during the maintaining phase is particularly significant because, during this phase, the AHAP only requires the hand to securely manipulate objects without imposing specific grip requirements. To ensure the secure handling of an object, the sum of all contact forces and the resulting friction forces must balance to zero. Given this principle, the challenges of the 3D-printed hands to succeed in the maintaining phase suggests potential issues such as insufficient friction, inadequate grip force, or geometric/kinematic limitations preventing the effective reorientation of contact forces. This suggests that the factors contributing to the lower performance of the 3D-printed prosthetic hands are rooted in their design or construction.

We believe that the primary challenge in the development of 3D-printed prostheses is balancing cost-effectiveness with the quality of materials and components. This often necessitates making strategic choices to maintain affordability but can compromise functionality, as shown in the AHAP maintaining scores. For instance, opting for cost-effective motors often compromises their achievable torque output, which directly impacts the prosthetic’s ability to securely hold and manipulate objects, thereby contributing to lower maintaining scores. Additionally, we acknowledge that the level of dimensional precision achievable by a 3D printer (how close the printed object’s dimensions are to the intended dimensions) may not always match that of mass-produced commercial prostheses. These dimensional differences can have implications for the fit and functionality of the prosthesis, underscoring the need for ongoing improvements in 3D printing technology. Durability and strength are also crucial, as commercial prostheses are designed for more rigorous use and are constructed from tested robust materials. We observed that the 3D-printed hands were limited in strength, being unable to support the weight of certain objects (skillet lid, wooden blocks with rope, and skillet). However, we also see opportunities for impactful enhancements through the cost-effective addition of certain features. For example, in our observations, the slipping of certain objects (large marker, small marker, and golf ball) from the HACKberry hand was not due to a lack of grip strength but rather to insufficient friction between the hand and the manipulated object. Consequently, adding rubberized grips to the fingers and palm or even offering rubberized gloves similar to those used to protect and provide friction for many CPCA hands could be a relatively simple yet effective method to significantly enhance the usability of the prosthesis. Another notable example is the difference in transmission mechanisms of the CPCA compared to the 3D-printed hands. The CPCA hands all typically have locking mechanisms with low backlash, so that when power is turned off the hands still hold their position, a useful feature for conserving battery as well as providing additional mechanical stability in the joint. None of the 3D-printed options that we examined currently employ these mechanisms and this may be another key feature that could be explored in future versions of 3D-printed hands to improve their maintaining scores.

While this study offers valuable insights into the performance of modern prosthetic hands, it does have limitations. The sample size for our study was limited to a specific subset of CPCA and 3D-printed prosthetic hands. For CPCA hands, this focus was primarily dictated by the challenges associated with acquiring these devices, often related to their high costs. Furthermore, there is a vast array of 3D-printed hands available, and the ones we tested do not encompass all existing models. Our selection was based on a limited subset chosen for the availability of documentation and open-access print files. It would be beneficial for future research to include a broader range of prosthetic hand technologies, especially those utilizing emerging designs and materials such as Unlimited Tomorrow and Open Bionics, as they are CPCA hands that were initially released as 3D-printed hands and were later refined and transitioned to commercially available devices [32]. Unfortunately, their cost, programming interfaces, and limited availability excluded them from this study. Furthermore, it may also be of interest to examine novel design concepts such as underactuated or soft prostheses as such systems are beginning to emerge in the prosthetics field.

Additionally, we acknowledge certain limitations in the AHAP [14]. Specifically, the grasping phase criteria may under-represent actual dexterity, as the strict guidelines for a correct grip do not always align with practical, clinical scenarios where a variety of secure grasping methods could be employed to manipulate an object. Additionally, the AHAP does not distinguish between grasping pressures, potentially allowing hands with superior grip strength to achieve a higher maintaining score by simply applying high gripping forces. In some real-world activities, performance is not necessarily constrained to the machine interacting with an object, but rather human-machine interactions can play a large role. That is, the choice of prosthetic control system, and the user’s skill in modulating grasping forces all can play a factor during many activities of daily living that may require manipulation of more fragile objects. Finally, the AHAP scores hand performance on an ordinal scale, which constrains the range of statistical inference methods that can be applied and necessitates the use of non-parametric methods. These methods, while appropriate, typically offer less statistical power compared to a parametric alternative. As a result, some grasps that may appear to differ greatly in their respective scores may not be considered significantly different by the Nemenyi Test. For example, during our testing we noted variances in the performance of the pulp pinch grip; however, these differences could not be statistically confirmed as significant through the Nemenyi Test. This suggests a need for further investigations, as it could reveal specific design enhancements necessary to improve the functionality of 3D-printed prosthetic hands.

Given that our findings demonstrate CPCA prostheses achieved significantly higher GAS scores than the 3D-printed hands, it suggests that these two types of devices may currently be best suited for distinct end uses. CPCA prostheses are evidently more practical for everyday use, as they have a higher technology readiness level given their ability to achieve a wider range of grip positions and superior performance in securely manipulating objects. Conversely, 3D-printed prosthetic devices, despite their limitations in grip capabilities, present a significantly more affordable option. With production costs as low as $19USD [9], they stand in stark contrast to the often prohibitive expenses associated with CPCA devices, which often exceed $20,000USD [7, 8]. Furthermore, the accessibility of 3D-printed prosthetic technology supports research to be conducted in labs of most funding levels, fostering a more inclusive research environment. This affordability is complemented by the technology’s capability for rapid prototyping, allowing researchers to quickly design, print, and test different prosthetic components, accelerating the pace of innovation and development. Open-source 3D-printed devices also benefit from non-proprietary firmware, allowing for more control over their devices. Additionally, researchers can tailor designs to fit various anatomical needs or specific user preferences, a crucial aspect in studying prosthetic functionality and comfort. Our study underlines the need for ongoing research and development in prosthetic technology, aiming to bridge the gap between affordability and functionality. Nevertheless, the potential of 3D printing in this field is immense, and recognizing these limitations through this and subsequent research will contribute to refining the design and functionality of both 3D-printed and CPCA prosthetic devices.

It is also important to note that the AHAP offers an important benchmark to assess the grasping capabilities of prosthetic hands; however, an individual user’s choice to use or abandon a prosthesis system arises from a complex, nuanced, and individualized combination of factors which include grasping performance among many others. These may include considerations not captured in such benchmark testing, such as the device’s ability to offer functional benefits during activities of daily living that offset the drawbacks of wearing the system, such as discomfort from socket and harnessing, temperature and sweat inside the socket, additional weight on the body, and numerous psychosocial factors, among others. Additionally, the suspension, support, and stabilization of the prosthesis on the residual limb can greatly affect the consistency and ease of use controlling the device, directly impacting the benefits offered by the grasping capabilities of the hand. Building on this work, it is important that further research compares 3D-printed and CPCA devices using common assessment tools and continues capturing this multitude of information that affects the real-world functional outcomes of a prosthesis prescription. This will inevitably require considering the needs of individuals using upper-limb prostheses, recruiting participants and prosthetic fittings to allow the examination of various hands/terminal devices, and careful consideration in designing experiments to control for construct validity given the multitude of complex factors affecting successful prosthetic outcomes.

## Electronic supplementary material

Below is the link to the electronic supplementary material.

Supplementary Material 1

Supplementary Material 2

## Data Availability

The datasets used and/or analysed during the current study are available in the supplementary information.

## References

[1] Armstrong TW, Williamson MLC, Elliott TR, Jackson WT, Kearns NT, Ryan T. Psychological distress among persons with upper extremity limb loss. Br J Health Psychol. 2019;24(4):746–763. 10.1111/bjhp.12360 10.1111/bjhp.12360 30941874

[2] Sheehan TP, Gondo GC. Impact of limb loss in the United States. Phys Med Rehabil Clin N Am. 2014;25(1):9–28. 10.1016/j.pmr.2013.09.007. 10.1016/j.pmr.2013.09.007 24287236

[3] Ma VY, Chan L, Carruthers KJ. The incidence, prevalence, costs and impact on disability of common conditions requiring Rehabilitation in the US: stroke, Spinal Cord Injury, traumatic Brain Injury, multiple sclerosis, osteoarthritis, rheumatoid arthritis, limb loss, and back Pain. Arch Phys Med Rehabil. 2014;95(5):986–95. 10.1016/j.apmr.2013.10.032. 10.1016/j.apmr.2013.10.032 PMC4180670 24462839

[4] Ziegler-Graham K, MacKenzie EJ, Ephraim PL, Travison TG, Brookmeyer R. Estimating the prevalence of limb loss in the United States: 2005 to 2050. Arch Phys Med Rehabil.2008;89(3):422–429. 10.1016/j.apmr.2007.11.005 10.1016/j.apmr.2007.11.005 18295618

[5] Dillon MP, Fatone S, Quigley M. Uncertainty with long-term predictions of lower-limb amputation prevalence and what this means for prosthetic and orthotic research. JPO J Prosthet Orthot. 2018;30(3):122. 10.1097/JPO.0000000000000191.

[6] Salminger S et al. Jul., Current rates of prosthetic usage in upper-limb amputees – have innovations had an impact on device acceptance? Disabil Rehabil. 2022;44(14):3708–3713. 10.1080/09638288.2020.1866684 10.1080/09638288.2020.1866684 33377803

[7] Wendo K et al. Open-source 3D printing in the prosthetic field—the case of upper limb prostheses: a review. Machines. 2022;10(6), Art. no. 6. 10.3390/machines10060413

[8] Getting to grips with bionic costs. Eureka. Accessed: Jan. 09, 2024. [Online]. Available: https://www.eurekamagazine.co.uk/content/technology/getting-to-grips-with-bionic-costs/

[9] Cabibihan J-J et al. Suitability of the openly accessible 3D printed prosthetic hands for war-wounded children. Front Robot AI. 2021;7. Accessed: Jan. 08, 2024. [Online]. Available: https://www.frontiersin.org/articles/10.3389/frobt.2020.594196 10.3389/frobt.2020.594196 PMC7830517 33501353

[10] Williams W. Bionic hand price list, bionics for everyone. Accessed: Aug. 23, 2023. [Online]. Available: https://bionicsforeveryone.com/bionic-hand-price-list/

[11] Llop-Harillo I, Pérez-González A, Andrés-Esperanza J. Grasping ability and motion synergies in affordable Tendon-Driven Prosthetic hands controlled by able-bodied subjects. Front Neurorobotics. 2020;14:57. 10.3389/fnbot.2020.00057. 10.3389/fnbot.2020.00057 PMC7480172 32982713

[12] Kannenberg A, Lundstrom R, Hibler KD, Johnson SS. Differences in two multiarticulating myoelectric hands for facilitating activities of daily living in individuals with transradial amputation: a cross-sectional study. JPO J Prosthet Orthot. 2023;35(1):38. 10.1097/JPO.0000000000000411.

[13] Belter JT, Segil JL, Dollar AM, Weir RF. Mechanical design and performance specifications of anthropomorphic prosthetic hands: a review. J Rehabil Res Dev. 2013;50(5):599. 10.1682/JRRD.2011.10.0188. 24013909 10.1682/jrrd.2011.10.0188

[14] Llop-Harillo I, Pérez-González A, Starke J, Asfour T. The anthropomorphic hand assessment protocol (AHAP). Robot Auton Syst. 2019;121:103259. 10.1016/j.robot.2019.103259.

[15] Siegel JR, Battraw MA, Winslow EJ, James MA, Joiner WM, Schofield JS. Review and critique of current testing protocols for upper-limb prostheses: a call for standardization amidst rapid technological advancements. Front Robot AI. 2023;10. Accessed: Nov. 14, 2023. [Online]. Available: https://www.frontiersin.org/articles/10.3389/frobt.2023.1292632 10.3389/frobt.2023.1292632 PMC10684749 38035123

[16] i-Limb^®^. Quantum bionic hand. Ossur.com. Accessed: Jan. 05, 2024. [Online]. Available: https://www.ossur.com/en-us/prosthetics/arms/i-limb-quantum

[17] ma00001a_i. -limb_ultra_datasheet.pdf. Accessed: Feb. 27, 2024. [Online]. Available: https://training.touchbionics.com/pdfs/ma00001a_i-limb_ultra_datasheet.pdf

[18] i-Limb_Quantum_Catalog_en-us_Q4-22.pdf. Accessed: Feb. 27, 2024. [Online]. Available: https://media.ossur.com/ossur-dam/image/upload/product-documents-global/i-Limb_Quantum_Catalog_en-us_Q4-22.pdf

[19] bebionic Hand. EQD | The most lifelike prosthetic hand. Accessed: Jan. 05, 2024. [Online]. Available: https://www.ottobock.com/en-us/product/8E70

[20] 14112_bebionic_user_guide_lo.pdf. Accessed: Jan. 31, 2024. [Online]. Available: https://www.ottobockus.com/media/local-media/prosthetics/upper-limb/files/14112_bebionic_user_guide_lo.pdf

[21] 127579-1-en_master-01-1704w_2508822.pdf. Accessed: Jan. 05. 2024. [Online]. Available: https://media.ottobock.com/_web-site/prosthetics/upper-limb/bebionic-hand/files/127579-1-en_master-01-1704w_2508822.pdf

[22] Ability Hand™,, Accessed PSYONIC. Jan. 12, 2024. [Online]. Available: https://www.psyonic.io/ability-hand

[23] PSYONIC + User + Manual + 2. 24 + 1.9.2024.pdf. Accessed: Feb. 27, 2024. [Online]. Available: https://static1.squarespace.com/static/61229f1b98e6c829c570bace/t/659ec6d3caa57e12edfa3b46/1704904420631/PSYONIC+User+Manual+2.24+1.9.2024.pdf

[24] HACKberry, Hand. HACKberry. Accessed: Jan. 12, 2024. [Online]. Available: https://www.exiii-hackberry.com

[25] Brenneis DJA, Dawson MR, Pilarski P. Development of the Handi hand: an inexpensive, multi-articulating, sensorized hand for machine learning research in myoelectric control; 2017. Accessed: Jan. 31, 2024. [Online]. Available: https://www.semanticscholar.org/paper/DEVELOPMENT-OF-THE-HANDI-HAND%3A-AN-INEXPENSIVE%2C-HAND-Brenneis-Dawson/598e0dc73d20c3fb4c78bdc6ba2a7ffe92c8e2f3

[26] Dawson M, Sherstan C, Carey J, Hebert J, Pilarski P. Development of the Bento arm: an improved robotic arm for myoelectric training and research; Aug. 2014. 10.13140/2.1.3118.4640.

[27] Battraw MA, Young PR, Joiner WM, Schofield JS. A multiarticulate pediatric prosthetic hand for clinical and research applications. Front Robot AI. 2022;9. Accessed: Jan. 31, 2024. [Online]. Available: https://www.frontiersin.org/articles/10.3389/frobt.2022.1000159 10.3389/frobt.2022.1000159 PMC9651148 36388251

[28] Llop-Harillo I, Pérez-González A. System for the experimental evaluation of anthropomorphic hands. Application to a new 3D-printed prosthetic hand prototype. Int Biomech. 2017;4(2):50–59. 10.1080/23335432.2017.1364666

[29] Mann HB, Whitney DR. On a test of whether one of two random variables is stochastically larger than the other. Ann Math Stat. 1947;18(1):50–60. 10.1214/aoms/1177730491

[30] Armstrong RA. When to use the Bonferroni correction. Ophthalmic Physiol Opt J Br Coll Ophthalmic Opt Optom. 2014;34(5):502–508. 10.1111/opo.12131 10.1111/opo.12131 24697967

[31] Friedman M. The use of ranks to avoid the assumption of normality implicit in the analysis of variance. J Am Stat Assoc. 1937;32(200):675–701. 10.1080/01621459.1937.10503522

[32] Nemenyi P. Distribution-free multiple comparisons. Accessed Jan. 12, 2024. [Online]. Available: https://search.library.ucdavis.edu

