# Supplementary material for: A performance evaluation of commercially available and 3D-printable prosthetic hands: a comparison using the anthropomorphic hand assessment protocol
Source: BMC Biomed Eng. 2024 Dec 2;6:11. doi: 10.1186/s42490-024-00086-w (PMC11610161; doi:10.1186/s42490-024-00086-w)
Supplement: Supplementary file 2 — Supplementary Material 2 [file 42490_2024_86_MOESM2_ESM.docx]

The supplementary material contains an excel file that has separate sheets for the raw data from each prosthesis tested in this paper. There are 10 separate sheets:

Raw Data:

1. Psyonic Ability Hand Raw Data
2. Ossur i-Limb Quantum Raw Data
3. BeBionic V3 Raw Data
4. BEAR PAW Raw Data
5. HANDi Hand Raw Data
6. HackBerry Hand Raw Data
7. IMMA Raw Data
8. Dextrus Raw Data
9. InMoov Raw Data
10. LimbitLess Raw Data
